# Differential mortality in Iran

**DOI:** 10.1186/1478-7954-5-7

**Published:** 2007-07-28

**Authors:** Ardeshir Khosravi, Richard Taylor, Mohsen Naghavi, Alan D Lopez

**Affiliations:** 1 School of Population Health University of Queensland, Brisbane, Australia; 2 Ministry of Health & Medical Education, Tehran, Iran

## Abstract

**Background:**

Among the available data provided by health information systems, data on mortality are commonly used not only as health indicators but also as socioeconomic development indices. Recognizing that in Iran accurate data on causes of death were not available, the Deputy of Health in the Ministry of Health and Medical Education (MOH&ME) established a new comprehensive system for death registration which started in one province (Bushehr) as a pilot in 1997, and was subsequently expanded to include all other provinces, except Tehran province. These data can be used to investigate the nature and extent of differences in mortality in Iran. The objective of this paper is to estimate provincial differences in the level of mortality using this death registration system.

**Methods:**

Data from the death registration system for 2004 for each province were evaluated for data completeness, and life tables were created for provinces after correction for under-enumeration of death registration. For those provinces where it was not possible to adjust the data on adult deaths by using the Brass Growth Balance method, adult mortality was predicted based on adult literacy using information from provinces with reliable data.

**Results:**

Child mortality (risk of a newborn dying before age 5, or _5_q_0_) in 2004 varied between 47 per 1000 live births for both sexes in Sistan and Baluchistan province, and 25 per 1000 live births in Tehran and Gilan provinces. For adults, provincial differences in mortality were much greater for males than females. Adult mortality (risk of dying between ages 15 and 60, or _45_q_15_) for females varied between 0.133 in Kerman province and 0.117 in Tehran province; for males the range was from 0.218 in Kerman to 0.149 in Tehran province. Life expectancy for females was highest in Tehran province (73.8 years) and lowest in Sistan and Baluchistan (70.9 years). For males, life expectancy ranged from 65.7 years in Sistan and Baluchistan province to 70.9 years in Tehran.

**Conclusion:**

Substantial differences in survival exist among the provinces of Iran. While the completeness of the death registration system operated by the Iranian MOH&ME appears to be acceptable in the majority of provinces, further efforts are needed to improve the quality of data on mortality in Iran, and to expand death registration to Tehran province.

## Background

To adequately measure population health a health information system is essential [1]. The main rationale for collecting routine data on population health is to provide information and evidence for designing and assessing health programs and to ensure that their objectives are being met [2,3]. Such data might also be used to generate or support observations about population health transition [2]. Among the available data generated by health information systems, data on mortality are the most commonly used, not only as indicators of health development, but also as broader measures of socioeconomic development.

Most, if not all countries possess legislation for vital registration systems to collect mortality data to generate various summary measures of population health [3]. However, a functioning vital registration system which yields valid and timely data on mortality requires considerable resources such as skilled manpower and technology, as well as processes to ensure that death data are reported, validated and used [4]. Developing countries usually do not have complete death registration and to estimate the level and trend of mortality based on available data, various demographic methods (e.g. Brass Growth Balance method) are used to adjust the data, particularly data on adult mortality [3,5].

There are various standard criteria that have been used elsewhere to assess the completeness and data quality of a death registration system [3,6]. Differential mortality information, (e.g. by age, sex, ethnicity, occupational category, place of residence) is a key output of a functioning death registration system [7]. Health decision makers are interested in the comparative health status of various segments of the population, and in different regions, to better target health programmes. Over the past three decades, research has been carried out to establish differential mortality patterns according to socioeconomic status (e.g. social class, income, ethnicity) and geography (e.g. rural and urban areas), based on data from various mortality information systems [7,8]. However, given the problems of death registration systems in developing countries, the majority of these studies have been conducted in developed countries. The few studies that have been carried out in developing countries are generally based on child mortality from health or demographic surveys [7,9].

In Iran there are three climatic zones; (a) arid/semi-arid regions, (b) mountainous extensions and (c) the Caspian region. Administratively, the country is divided into 30 provinces with different levels of socioeconomic development. The variations in socioeconomic condition, climate and the environment across the country may imply differences in the level and distribution of health-related indices between regions and provinces [10]. Table 1 summarizes the variation in socioeconomic conditions across provinces according to various data sources. If socioeconomic status and geographic conditions are also strong predictors of mortality in Iran, as elsewhere, one would expect corresponding provincial differences in survival. However, this has not yet been established.

**Table 1:** Selected population and socioeconomic indicators by provinces (sorted by total % literate), Iran, 2004

**Province**	**Population^a^**	**U/R^b^**	**TFR^c^**	**Literacy rate % (15+)^d^**		**Unemployment rate % (10+)^e^**		**GDP per capita (Iranian Rial) (2000–2003) ^f^**
					
				**Male**	**Female**	**Male**	**Female**	
Tehran	12150742	6.5	1.6	91	85	15	30	19752051
Semnan	552442	2.8	2.2	87	80	13	19	12087729
Esfahan	4237512	4.2	1.7	87	77	14	12	12753060
Yazd	923642	4	2.5	86	77	13	9	12508297
Qom	1026711	15.5	2.6	86	74	16	27	9459869
Fars	4342423	1.4	1.7	86	76	19	23	9469063
Qazvin	1096453	1.8	1.8	85	74	12	7	12751667
Bushehr	853669	1.7	2.3	84	73	19	21	21701172
Mazandaran	2850625	1.1	1.7	84	74	14	22	11743883
Gilan	2425416	1.1	1.4	84	74	15	25	9316561
Khuzestan	4173118	2.1	2.6	83	69	27	33	28214087
Khorasan	6239346	1.6	2.4	82	72	14	21	8949320
Markazi	1300107	2	1.7	82	70	14	16	16214755
East Azerbaijan	3587976	1.9	2.1	81	66	12	12	10419474
Golestan	1644254	0.9	2.3	81	67	16	21	8190491
kerman	2322886	1.3	2.4	81	73	22	39	10831166
Hamedan	1720658	1.3	1.9	80	66	16	24	8602788
Ilam	503658	1.3	2	80	68	29	37	11020026
Char Mahal & Bakhtiari	870216	1.1	2.2	80	65	19	20	7167414
Kohgilooye & Boyer-Ahmad	618794	0.8	2.3	79	64	21	44	56820967
Kermanshah	1904289	2.1	1.8	79	64	19	22	6737277
Zanjan	989166	1.2	2	78	64	10	10	8252266
Ardebil	1312200	1.1	2	78	58	18	18	7742049
Lorestan	1640896	1.5	1.9	78	65	21	22	7026604
West Azerbaijan	2838014	2.6	2.7	77	58	13	12	6853321
Hormorzgan	1263382	0.8	2.7	77	64	27	50	12699599
Kordistan	1454881	1	1.9	75	54	18	15	5987269
Sistan & Baluchistan	2164233	0.9	4.1	65	46	27	53	4874351

Total country	67007709	1.9	2	82	69	17	24	13681914

^a ^Population in 2004

^b ^Ratio of population in urban areas to rural areas

^c ^Total fertility rate in 2000 based on the DHS 2000 [23]

^d ^Literacy rate for age 15 and above according to the Statistical Centre of Iran (SCI) [25]

^e ^Unemployment rate for age 10 and above according to DHS 2000 [23]

^f ^Average Gross Domestic Product (Iranian Rial) (2000–2003) [27]

The objective of this paper is to estimate the level of mortality in terms of probability of child (_5_q_0_) and adult (_45_q_15_) death, and life expectancy at birth, among the various provinces in Iran, and also to determine to what extent these mortality indices are consistent with the socioeconomic characteristics of provinces in Iran.

## Data Sources and adjustments

In Iran, two organizations, the National Organization for Civil Registration (NOCR) and the Ministry of Health and Medical Education (MOH&ME) currently operate death registration systems. The NOCR is legally responsible for the registration of four vital events (birth, death, marriage and divorce) [11]. It is generally agreed that of the four events registered by the NOCR, mortality data are the least complete and of the lowest quality [12]. Although data from 1995 show that this organization has made progress in capturing and registering deaths, there remains substantial delayed registration and inaccurate recording and reporting of the cause of death [13]. Thus, these data have not been widely used for analysing mortality in previous studies in Iran.

The MOH&ME (Deputy of Research and Technology) collected data on mortality during the period 1965–2001 [14,15]. The main aim of this data collection system was to obtain information on the structure of causes of death in Iran [16-19]. This data source was based on data on causes of death from cemeteries and was discontinued in 2002 with the introduction of a new death registration system by MOH&ME (Deputy of Health) [16-19].

**Figure 1:**
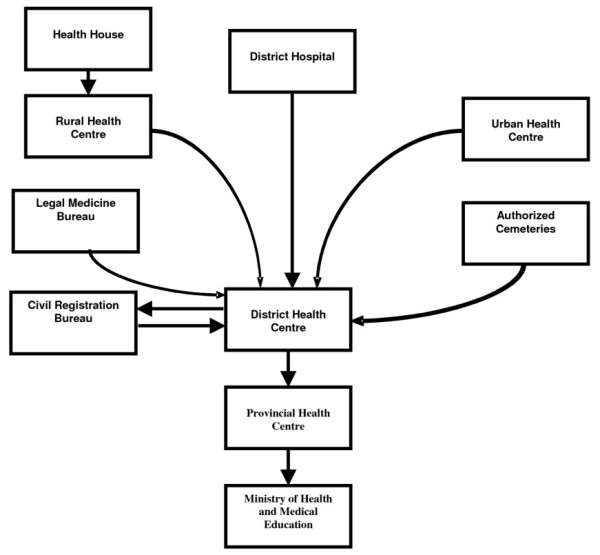
Flow of data through the death registration system operated by the Ministry of Health and Medical Education (Deputy of Health).

Recognizing that in Iran accurate data on causes of death were not available, the Deputy of Health in the MOH&ME initiated a new comprehensive program for death registration in order to improve the capacity of district health networks for registering deaths by age, sex, cause, and place of residence. This project started in one province (Bushehr) as a pilot in 1997 [20]. To improve the level and quality of registration, data from different sources (e.g. NOCR, cemetery, and hospital) have been integrated and cross-checked to remove duplication and to improve registration coverage. Moreover, registration is active, in that health workers at each health facility are responsible for identifying deaths among the population (see Figure 1) [13]. In 2001, a comparison with other sources (e.g. the Iranian NOCR and Deputy of Research and Technology of the MOH&ME) revealed that both completeness and classification of causes of death were better than other sources [13]. This system was then progressively implemented in several provinces in subsequent years and by 2006, 29 provinces were covered by the system. It is expected that all 30 provinces (including Tehran province) will covered be the end of 2007 (See Table 2).

**Table 2:** Number of deaths reported by provinces and population covered by the Iranian MOH&ME (Deputy of Health) death registration system, Iran, 1999–2004

		**Population covered**		
			
**Year of data**	**Provinces covered**	**Number**	**% of total**	**Number of deaths reported**
1999	4	5509915	9	18939
2000	10	16737094	26	66846
2001	18	36775017	57	146159
2002	18	38271475	60	168728
2003	23	48288732	73	212227
2004	29	54815786	82	240786

Source: Iranian Ministry of Health and Medical Education [13, 14, 20, 47]

To estimate the key mortality indices (_5_q_0_, _45_q_15 _and life expectancy at birth) by province, data from the death registration system operated by the MOH&ME (Deputy of Health) in 2004 by age, sex and province were obtained. This was the latest year for which data were available. Data on deaths by age, sex and cause were available for 29 (out of 30) provinces, the exception being Tehran province (12 million people) which was not yet covered by this death registration system. To estimate the level of mortality in Tehran province, the latest available data on mortality (2001) from the death registration system operated by the MOH&ME (Deputy of Research and Technology) were used (see below).

Khorasan province was divided into three provinces in 2004. However, since data on population size and socioeconomic status (e.g. literacy and GDP) for the new provinces were not available for this study, we have aggregated the data for these provinces into Khorasan province.

To estimate mortality rates, midyear population data by age and sex for each province are required as denominators. As the last census was carried out in Iran in 1996, provincial population data for 2004 were estimated as follows. At the beginning of each year a census of households is conducted in rural areas by health workers from local rural health units. Population data for those rural areas not covered by health units are collected by mobile health worker teams. We collated these sources to obtain population estimates by age and sex for rural areas of Iran in 2004 [21].

For urban populations, as for rural areas, a census of households covered by urban health units is carried out each year [21]. For several large cities (capital cities of provinces) for which the coverage of the health network is not complete, populations by age and sex were estimated. To do this, the total population size of each city in 2004 was first obtained from the Statistical Centre of Iran, based on trends in population growth rates between 1991 and 1996 (based on the 1991 inter-censal survey and the census of 1996) [22]. The proportional age-sex distribution of the population for each city was then obtained from the Iranian Demographic and Health Survey in 2000 and was used to estimate the population of males and females in 5 year age groups in urban areas in 2004 [23].

As the data from the death registration system were not complete, we needed to adjust them to estimate mortality rates and create life tables for each province [24]. Mortality data for children and adults were adjusted separately. Since there are no previous estimates of _5_q_0 _by province, we used the latest available estimates of infant mortality (_1_q_0_) for each province in 2001 obtained from the Statistical Centre of Iran (SCI), based on the results of the multi-round Population Growth Survey (1998–1999), using data on children ever-born and children surviving and analysed according to the Brass method [25,26].

In order to assess the socioeconomic status of each province, Gross Domestic Product (GDP) and Literacy were used. The last available data on GDP per capita by province, estimated by the Statistical Centre of Iran, was for the period 2000–2003. The annual average over this period was used for this study [27]. Literacy levels (in %) for the population aged 15 years and over, by province, were obtained from the Population Data Sheet of Iran in 2001, which in turn was based on the results of the Labour Force Survey of 2001 carried out by the SCI [28]. In this survey a literate person was defined as: "Anyone who can read and write a simple text in Farsi or any other language irrespective of formal certification" [29].

## Methods

Generally, in situations where mortality data, particularly data from death registration, are poor, there are two approaches to correct the data: indirect and direct [30]. There are various indirect demographic methods available for estimating the completeness of death registration data (e.g. Brass Growth Balance method, Bennett-Horiuchi method) [31]. In these techniques, the age distribution of reported deaths is compared with the age distribution of the population, under certain assumptions [32]. Completeness can also be assessed directly using the "capture-recapture" approach. That is, deaths reported in an independent survey of mortality are compared to deaths reported in the death registration system for the same population, from which unmatched and unrecorded deaths can be identified and estimated [30]. This is a more expensive means to assess completeness since a sufficiently large population sample is required.

The first analytical step in this study was the computation of age-specific death rates by province. Firstly, data on deaths by 5 year age groups were reviewed. Those deaths for which there was no information on age (1,533 deaths) and sex (637 deaths) were proportionately redistributed across age and sex categories based on observed data. In addition, data on 6,245 deaths (out of 240,754 deaths in 2004) where the place of usual residence (districts) of the deceased was unknown were proportionally redistributed across all provinces.

To correct for under-enumeration of deaths, the following methods were used.

### Child deaths (0–5 years)

The latest available estimates of infant mortality (_1_q_0_) by province were for 2001. To estimate child mortality in 2004, _1_q_0 _was first estimated for each province in 2004 based on the estimated decline in the national infant mortality rate over the period 2001–2004 [33]. Provincial estimates of infant mortality in 2004 were adjusted to be consistent with _1_q_0 _at the national level [33]. Finally, we converted _1_q_0 _to _5_q_0 _based on the ratio of _1_q_0 _to _5_q_0 _in the Iranian Demographic Health Survey (DHS 2000) for each sex separately [23].

### Adult deaths (5 years and over)

The Brass Growth Balance method was first applied to provincial data to correct for undercount of adult deaths. This method is based on the general balancing equation in demography, whereby the death rate in any population has a defined relationship with the birth rate and the rate of growth of that population [34]. In this method it is assumed that the population is stable and closed to migration and that the partial (by age) birth rates have a linear relationship with partial death rates. Application to provincial mortality data revealed that for several provinces, the Brass Growth Balance method was inapplicable (that is, the correction factors based on the regression of partial birth and death rates were either less than 0.9 or higher than 1.5, implying a level of completeness of registration of greater than 110% or less than about 60%, a level below which it is recommended that the method not be used [30]. Undoubtedly, the reason for this is violation of the assumption of a closed population. To proceed, provinces were grouped into three categories: (a) provinces with estimated completeness of registration between 0.9 and 1.1 (i.e. registration was considered complete, or nearly so) (b) provinces for which the Brass Growth Balance method provided plausible estimates of completeness, and (c) provinces for which the Brass Growth Balance method appeared not to work.

Next, we checked the plausibility of the adjusted _45_q_15 _based on this categorization. To do this we first predicted _45_q_15 _from _5_q_0 _based on the Modified Logit Life Table System (MLLTS) [35]. This model predicts what the expected level of _45_q_15 _would be based on an estimate of _5_q_0. _The predicted level of _45_q_15 _for each province was computed for ± 3 standard deviations of the distribution of values of _45_q_15_, as well as the mean [35,36]. The adjusted _45_q_15 _in groups (a) and (b) were then plotted against predicted _45_q_15 _(the mean values of MLLTS). We also examined the scatter plot of the adjusted values of _45_q_15 _in groups (a) and (b) against literacy levels using regression models.

These analyses revealed that for some provinces grouped under (a) and (b), the adjusted values of _45_q_15 _were clearly outliers and not plausible. Furthermore, for those provinces in groups (a) and (b) for which _45_q_15 _values were not plausible, estimated life expectancies at birth compared to the national level and other similar provinces were substantially higher (e.g. between 71 and 72 years for males and between 74 and 76 for females).

On the other hand, in several provinces for which the Brass Growth Balance method appeared not to work, and which were categorized into group (c), the calculated value of _45_q_15 _and estimated life expectancy from vital registration appeared plausible, particularly for males. Some of these provinces have had operational death registration systems for some time (e.g. Fars, Kerman and Khorasan). As a result, we added the implausible values from groups (a) or (b) to group (c), and the plausible values from group (c) into group (a). The final categorization of provinces into the 3 groups is shown in Table 3.

**Table 3:** Categorization of provinces based on completeness of data on adult deaths, Iran, 2004

**Province**	**Females**				**Males**			
	
	**Completeness**	**Registered _45_q_15_**	**Adjusted _45_q_15_**	**category**	**Completeness**	**Registered _45_q_15_**	**Adjusted _45_q_15_**	**category**
**Ardebil**	0.67	0.065	0.095	c	1.28	0.142	-	c
**Bushehr**	0.93	0.108	0.115	a	1.09	0.186	0.186	a
**Charmahal & Bakhtiari**	1.08	0.092	-	c	1.41	0.144	-	c
**East Azerbaijan**	0.86	0.091	-	c	0.74	0.169	-	c
**Esfahan**	0.76	0.064	-	c	0.91	0.141	-	c
**Fars**	0.95	0.093	0.097	a	1.25	0.211	0.211	a
**Gilan**	0.79	0.092	0.115	b	1	0.191	0.191	a
**Golestan**	0.71	0.087	0.119	b	0.92	0.199	0.215	a
**Hamedan**	0.65	0.06	-	c	0.99	0.143	-	c
**Hormozgan**	0.86	0.097	0.112	b	0.96	0.19	0.196	a
**Ilam**	0.81	0.095	0.116	b	1.69	0.189	-	c
**Kerman**	0.99	0.122	0.123	a	1.49	0.244	0.244	a
**Kermanshah**	0.84	0.102	0.121	b	1.02	0.22	0.22	a
**Khorasan**	0.83	0.095	0.113	b	1.85	0.19	0.19	a
**Khuzestan**	0.72	0.083	0.114	b	0.74	0.151	0.197	b
**Kohgilooyeh & Boyer-Ahamad**	1.25	0.1	-	c	1.54	0.131	-	c
**Kurdistan**	0.68	0.083	0.119	b	1.3	0.154	-	c
**Lorestan**	0.77	0.09	0.116	b	1.59	0.201	0.201	a
**Markazi**	0.85	0.077	0.09	c	1.22	0.168	-	c
**Mazandaran**	0.37	0.091	-	c	0.65	0.091	-	c
**Qazvin**	0.88	0.095	0.107	b	1.11	0.168	-	c
**Qom**	0.59	0.054	-	c	0.59	0.091	-	c
**Semnan**	1.05	0.107	-	c	1.22	0.175	0.175	a
**Sistan & Baluchistan**	0.56	0.09	0.155	c	0.98	0.199	-	c
**Tehran**	0.43	0.061	0.135	c	0.49	0.142	-	c
**West Azerbaijan**	0.76	0.085	0.11	b	0.81	0.17	0.209	b
**Yazd**	1.08	0.073	-	c	0.98	0.158	-	c
**Zanjan**	1.04	0.084	-	c	1.75	0.179	-	c

a: provinces for which death registration was considered complete, or nearly so

b: provinces for which the Brass Growth Balance method provided plausible estimates of completeness,

c: provinces for which the Brass Growth Balance gave implausible estimates of completeness

**Figure 2:**
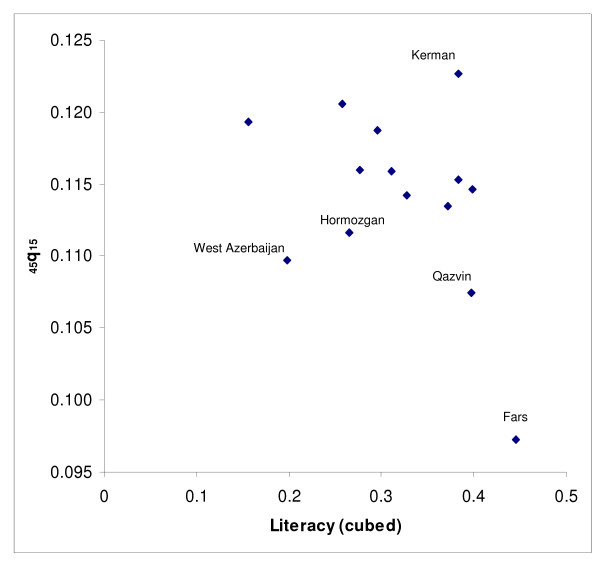
**Scatter plot of _45_q_15 _against literacy (cubed) for females, Iran, 2004**. Note: The scatter plot corresponds to those provinces in the a and b groups (provinces with plausible values of _45_q_15_).

**Figure 3:**
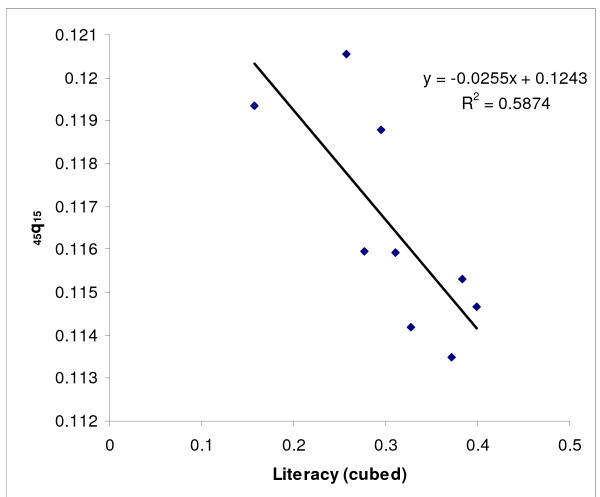
**Scatter plot and fitted line of _45_q_15 _against literacy (cubed) for females after censoring outlying data, Iran, 2004**. Note: The scatter plot corresponds to those provinces in the a and b groups (provinces with plausible values of _45_q_15_). While a linear fit is reasonable across the range of data values, an exponential or other non-linear form would better represent out of sample points.

**Figure 4:**
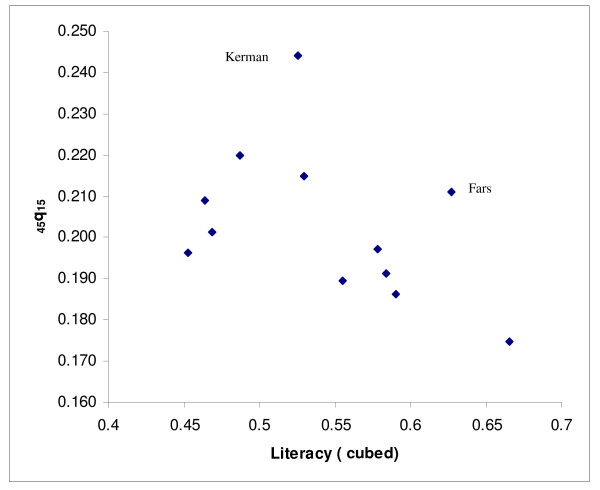
**Scatter plot of _45_q_15 _against literacy (cubed) for males, Iran, 2004**. Note: The scatter plot corresponds to those provinces in the a and b groups (provinces with the plausible values of _45_q_15_).

**Figure 5:**
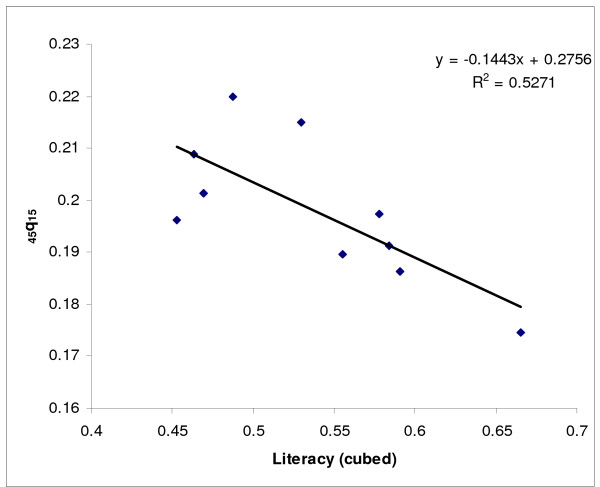
**Scatter plot and fitted line of _45_q_15 _against literacy (cubed) for males after censoring outlying data, Iran, 2004**. Note: The scatter plot corresponds to those provinces in the a and b groups (provinces with the plausible values of _45_q_15_). (see footnote to Figure 3).

Next, we modelled adult mortality level for provinces from groups (a) and (b) as a function of two socioeconomic indices (GDP/capita and % literacy) using linear regression. Since the predicted values of _45_q_15 _based on GDP per capita for the oil producing provinces were not plausible, only the relationship with literacy was used for all provinces. To do this, literacy data were first transformed to a cubic scale to increase predictive power. The plots of the residual of the predicted values of _45_q_15 _against literacy suggested that some values fell outside the 95% confidence limits: Fars, Qazvin, Hormzgan, Kerman and West Azerbaijan provinces for females, and Kerman and Fars for males. Since these values were clearly outliers, data from these provinces were censored and predictions were based on data from the remaining provinces (see Figures 2 &3 for females and Figures 4 &5 for males).

**Figure 6:**
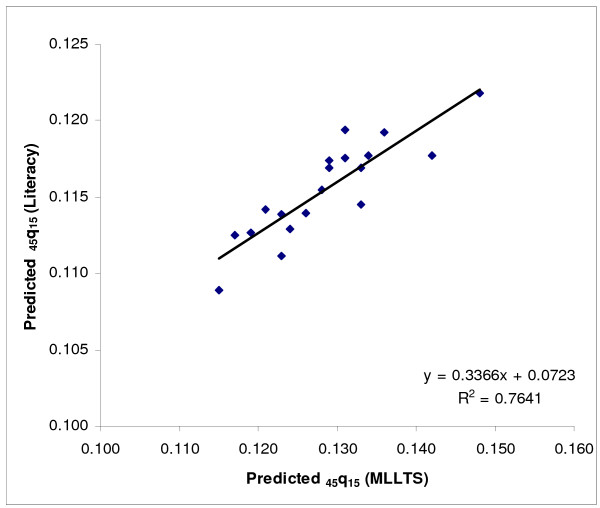
**Scatter plot and fitted line of predicted _45_q_15 _based on literacy and MLLTS, females, Iran, 2004**. MLLTS: Modified Logit Life Table System. Note: The scatter plot includes provinces for which the Growth Balance Brass method did not work and those that had their values censored in the regression model and where the values of _45_q_15 _were predicted based on literacy.

**Figure 7:**
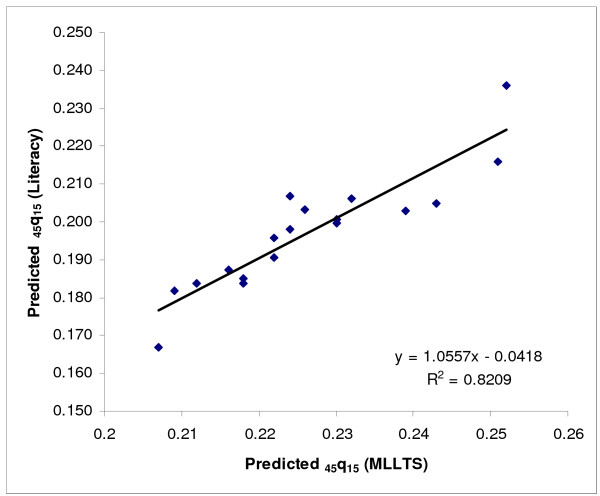
**Scatter plot and fitted line of predicted _45_q_15 _based on literacy and MLLTS, males, Iran, 2004**. MLLTS: Modified Logit Life Table System. Note: The scatter plot includes provinces for which the Growth Balance Brass method did not work and those that had their values censored in the regression model and where the values of _45_q_15 _were predicted based on literacy.

As a final check on the plausibility of the actual and predicted values of _45_q_15 _for all provinces, we then compared the predicted level of _45_q_15 _based on the MLLTS with the level predicted based on the basis of the literacy rate. Comparing the predicted _45_q_15 _based on literacy with _45_q_15 _as predicted by using the MLLTS (mean values) suggests that for those provinces for which the Brass Growth Balance method was inapplicable, literacy was a reasonable predictor of adult mortality for both males and females. Moreover, this method could be used for oil producing provinces (see Figures 6 &7).

As we had previously estimated _45_q_15 _at the national level for Iran (0.124 for females and 0.175 for males) [33], to ensure internal consistency of estimates we scaled the provincial values of this measure to be congruent with the estimated value for the national level. For those provinces where the registered levels were plausible, we did not make any adjustment. For all other provinces, where we predicted _45_q_15 _by either literacy or the Brass Growth Balance method, we scaled only those values so that the weighted _45_q_15 _matched exactly the estimated value for Iran. To check whether this led to any distortions, this adjustedset of _45_q_15 _was plotted against the predicted value from the MLLTS method. No discernible distortions were apparent.

Finally, to estimate the completeness of registered data on deaths for each province, the ratio of adjusted to registered values for both _5_q_0 _and _45_q_15 _were estimated. From the final estimates of _5_q_0 _and _45_q_15_, the full set of age-specific death rates and life tables for both sexes were constructed by using the Modified Logit Life Table System. Data were analysed using Stata version 9.2 and Excel 2003 [37].

## Results

### Completeness of data on mortality

Table 4 provides estimates of the completeness of child mortality data for provinces by sex (ratio of adjusted to registered _5_q_0_). Three provinces, Tehran (29%), Mazandaran (30%) and Lorestan (36%), have the lowest level of completeness of child mortality registration for both sexes. Completeness is highest in Qazvin (93%), Yazd (88%) and Semnan (79%). The results for Tehran are not surprising since the data on mortality for Tehran province were taken from cemetery registers (the Iranian MOH&ME – Deputy of Research and Technology) in 2001, which tend to have a low level of completeness.

**Table 4:** Estimated completeness of mortality data for children (<5) and adults (aged 15–60 years) by province and sex (sorted by completeness of adult deaths for both sexes), Iran, 2004

**Provinces**	**Completeness (%) of child death registration based on _5_q_0_**			**Completeness (%) of adult death registration based on _45_q_15_**		
	
	**Females**	**Males**	**Both**	**Females**	**Males**	**Both**
**Kerman**	71	81	76	92	112	102 (= 100)
**Kermanshah**	43	67	55	79	112	96
**Semnan**	69	89	79	90	100	95
**Bushehr**	73	72	73	87	100	94
**Fars**	70	79	74	76	112	94
**Khorasan**	79	73	76	77	100	89
**Qazvin**	95	92	93	77	100	89
**Gilan**	47	52	50	75	100	88
**Golestan**	39	65	52	69	104	87
**Hormorzgan**	77	80	79	77	97	87
**Lorestan**	32	40	36	71	100	86
**Ilam**	41	36	39	76	93	85
**East Azerbaijan**	54	67	61	72	96	84
**Sistan & Baluchistan**	48	64	56	69	95	82
**Zanjan**	78	67	72	66	97	82
**Markazi**	60	64	62	62	96	79
**West Azarbaijan**	54	67	60	66	91	79
**Yazd**	85	90	88	60	96	78
**Khuzestan**	53	68	60	67	86	77
**Char Mahal & Bakhtiari**	65	74	70	72	79	76
**Kohgilooye & Boyer-Ahm ad**	64	66	65	78	72	75
**Tehran**	28	30	29	52	95	74
**Kordistan**	44	53	49	64	80	72
**Esfahan**	59	69	64	53	87	70
**Ardebil**	49	43	46	50	77	64
**Hamedan**	61	77	69	48	80	64
**Mazandaran**	26	34	30	74	53	64
**Qom**	41	55	48	44	56	50

Table 4 also shows the estimated completeness of adult death registration for provinces according to the adjusted _45_q_15_. The results suggest that completeness for both sexes in provinces such as, Kerman, Kermanshah and Semnan, Bushehr and Fars, which have had longer experience in the operation of the Iranian MOH&ME death registration system, is comparatively high. The estimated values of _45_q_15 _for males based on registered data for Kerman, Kermanshah and Fars provinces are in fact higher than the adjusted data suggests. Two provinces, Qom (50%) and Mazandaran (64%), have the lowest completeness of registration of adult deaths for both sexes. Death registration is passive in both of these provinces, and death data are mainly collected from cemetery registers which may explain the relatively high under-recording of deaths.

### Estimation of child mortality by province in 2004

Table 5 shows estimates of _5_q_0 _by sex for provinces of Iran in 2004 (with 3 provinces amalgamated into Korasan). According to these estimates, Sistan and Baluchistan province had the highest _5_q_0 _(47 per 1000 live births) for both sexes, followed by Kurdistan (46 per 1000 live births) and Kohgilooye and Boyer-Ahmad (42 per 1000 live births). Tehran and Gilan provinces, with 25 child deaths per 1000 live births, had the lowest _5_q_0 _for both sexes, followed by Esfahan (26 per 1000 live births).

**Table 5:** Estimated _5_q_0 _per 1000 live births by province and sex (sorted by both sexes), Iran, 2004

**Province**	**_5_q_0_**		
	
	**Females**	**Males**	**Both**
**Tehran**	23	27	25
**Gilan**	23	27	25
**Esfahan**	24	28	26
**Yazd**	25	29	27
**Qazvin**	26	31	29
**Qom**	27	32	29
**Semnan**	27	31	29
**Fars**	28	32	30
**Khuzestan**	29	34	32
**Markazi**	30	34	32
**Mazandaran**	29	34	32
**Bushehr**	31	36	33
**East Azerbaijan**	31	35	33
**Char Mahal & Bakhtiari**	31	36	34
**Hormorzgan**	32	37	34
**Ardebil**	32	37	35
**Hamedan**	33	38	35
**Golestan**	33	38	36
**Kerman**	33	38	36
**Zanjan**	34	39	36
**Kermanshah**	34	40	37
**Lorestan**	35	41	38
**West Azarbaijan**	35	40	38
**Khorasan**	36	42	39
**Ilam**	37	43	40
**Kohgilooye & Boyer-Ahmad**	39	45	42
**Kordistan**	42	49	46
**Sistan & Baluchistan**	44	51	47

**National estimate [33]**	30	35	32

**Figure 8:**
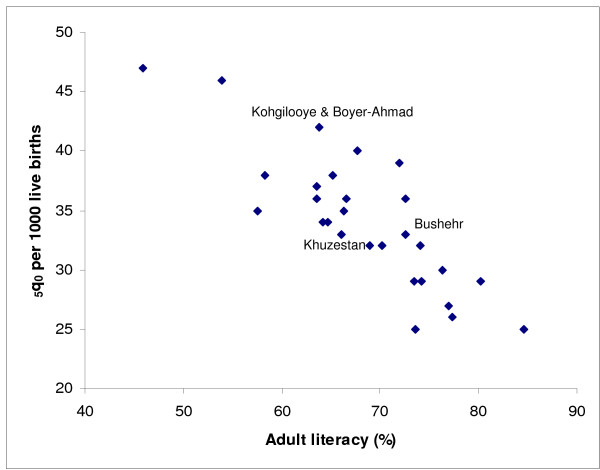
**Scatter plot of _5_q_0 _(both sexes) against literacy, Iran, 2004**. Note: Kohgilooye and Boyer-Ahmad, Khuzestan and Bushehr are the three oil producing provinces.

Figure 8 shows the estimated _5_q_0 _for 2004 for all provinces compared with the level of literacy of the population aged 15 years and above in 2001, according to the estimates from the Statistical Centre of Iran. Literacy appears to have an almost linear correlation with child mortality (_5_q_0_), confirming findings from other populations [38,39].

### Adult Mortality

Estimated probabilities of dying between the ages of 15 to 60 years (_45_q_15_) for each province based on registered, adjusted data for males and females are shown in Table 6. Adult mortality (_45_q_15_) among women is highest in Kerman (0.132), Sistan and Baluchistan (0.131) and Kermanshah (0.130) provinces and lowest in Tehran (0.117), Esfahan (0.121) and Yazd (0.122). Kerman province had the highest _45_q_15 _for males (0.218) followed by Sistan and Baluchistan (0.211) and Ilam (0.203) provinces. Tehran (0.149), Esfahan (0.162) and Qom (0.164) and Yazd (0.164) had the lowest _45_q_15 _for men.

**Table 6:** Estimated _45_q_15 _based on registered and adjusted data and life expectancy at birth by province and sex (sorted by life expectancy for both sexes), Iran, 2004

**Province**	**Registered _45_q_15_**		**Adjusted _45_q_15_**		**Life expectancy at birth (years)**	
	
	**Females**	**Males**	**Females**	**Males**	**Females**	**Males**
**Tehran**	0.061	0.142	0.117	0.149	73.82	70.77
**Yazd**	0.073	0.158	0.122	0.164	73.27	69.91
**Esfahan**	0.064	0.141	0.121	0.162	72.74	70.09
**Azvin**	0.095	0.168	0.123	0.167	73.18	69.54
**Qom**	0.054	0.091	0.123	0.164	73.03	69.53
**Semnan**	0.107	0.175	0.12	0.175	73.2	69.21
**Gilan**	0.092	0.191	0.123	0.191	73.48	68.73
**Mazandaran**	0.091	0.091	0.123	0.17	72.8	69.18
**Khuzestan**	0.083	0.151	0.125	0.176	72.68	68.86
**Markazi**	0.077	0.168	0.125	0.175	72.6	68.86
**Fars**	0.093	0.211	0.122	0.188	72.99	68.36
**East Azerbaijan**	0.091	0.169	0.126	0.177	72.44	68.69
**Charmahal & Bakhtiari**	0.092	0.144	0.127	0.181	72.33	68.52
**Hamedan**	0.06	0.143	0.126	0.179	72.36	68.36
**Bushehr**	0.108	0.186	0.124	0.186	72.52	68.19
**Ardebil**	0.065	0.142	0.129	0.185	72.22	68.19
**Zanjan**	0.084	0.179	0.127	0.184	72.07	68.04
**Golestan**	0.087	0.199	0.126	0.192	72.36	67.71
**Hormozgan**	0.097	0.19	0.127	0.196	72.33	67.69
**West Azerbaijan**	0.085	0.17	0.129	0.186	71.89	67.88
**Lorestan**	0.09	0.201	0.126	0.201	72.11	67.59
**Khorasan**	0.095	0.19	0.124	0.19	72.04	67.57
**Kermanshah**	0.102	0.22	0.13	0.196	71.97	67.55
**Kohgilooyeh & Boyer-Ahamad**	0.1	0.131	0.127	0.183	71.63	67.59
**Ilam**	0.095	0.189	0.126	0.203	71.86	66.92
**Kerman**	0.122	0.244	0.132	0.218	72.04	66.65
**Kurdistan**	0.083	0.154	0.13	0.193	71.23	66.85
**Sistan & Baluchistan**	0.09	0.199	0.131	0.211	70.91	65.92

**National level without Tehran province [33]**	0.072	0.159	0.124	0.175	71.19	68.72

These values compare with an estimated national level of adult mortality (_45_q_15_) of 0.124 for females and 0.175 for males (see Table 6) [33]. While the provincial differences are important, they are substantially less than what is observed in other developing countries [40].

**Figure 9:**
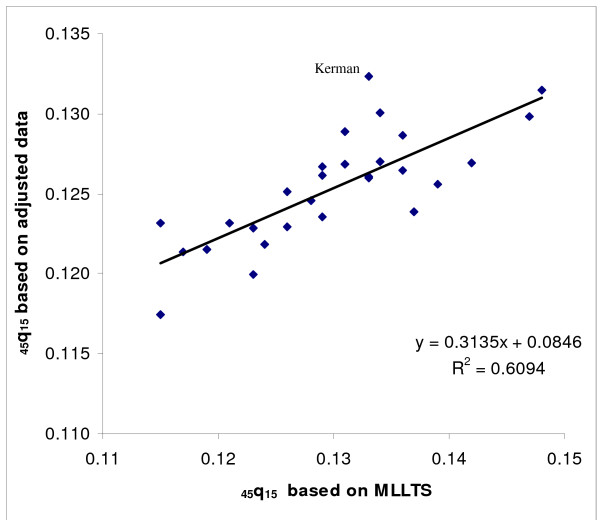
**Scatter plot and fitted line of estimated _45_q_15 _for all provinces and predicted by MLLTS, females, Iran, 2004**. MLLTS: Modified Logit Life Table System.

**Figure 10:**
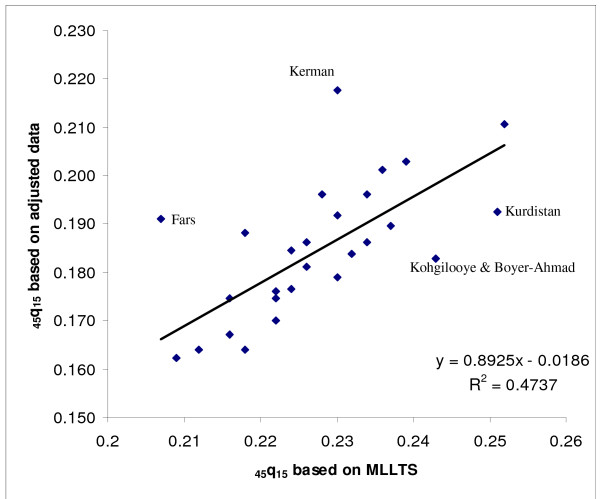
**Scatter plot and fitted line of estimated _45_q_15 _for all provinces and predicted by MLLTS, males, Iran, 2004**. MLLTS: Modified Logit Life Table System.

Figures 9 and 10 show scatter plots of our final estimates of _45_q_15 _against predicted values for this measure based on the MLLTS. Values based on adjusted data are more consistency with predicted values for females than for males. Figure 6b illustrates that, for males, four provinces (Kerman, Fars, Kurdistan and Kohgilooye & Boyer-Ahmad) are clear outliers. This might be due to the fact that child mortality in these provinces is comparatively high, and the MLLTS will exaggerate predicted _45_q_15 _in such cases. On the contrary, Kerman and Fars provinces, which had relatively high levels of adult mortality according to registered data, have low levels of child mortality and hence low predicted adult mortality levels. These aberrations suggest that the MLLTS should be used only as a broad check on the plausibility of predicted adult mortality levels using our methods.

### Life expectancy

Table 6 shows estimated life expectancy at birth by province for males and females in Iran in 2004. Not surprisingly, Sistan and Baluchistan province had the lowest life expectancy at birth for both females (70.9 years) and males (65.9 years) among all provinces. Tehran province had the highest life expectancy at birth for both females (73.8 years) and males (70.8 years). The value of this index for Iran (excluding Tehran province) was 71.2 years for females and 68.7 years for males. This range of values in life expectancy is comparatively small, and certainly much less than other countries such as Brazil or the United States, where mortality variations, particularly among males, are substantial [40].

## Discussion

This study provides comprehensive comparative estimates of mortality at the sub-national level in Iran. For the first time, age-specific death rates have been estimated at the provincial level of Iran based on empirical data. As data from the death registration system were not complete, different approaches such as the Brass Growth Balance Method and regression models based on literacy were used to correct the registered data on adult deaths. From the corrected mortality data, life tables were constructed for each province.

**Figure 11:**
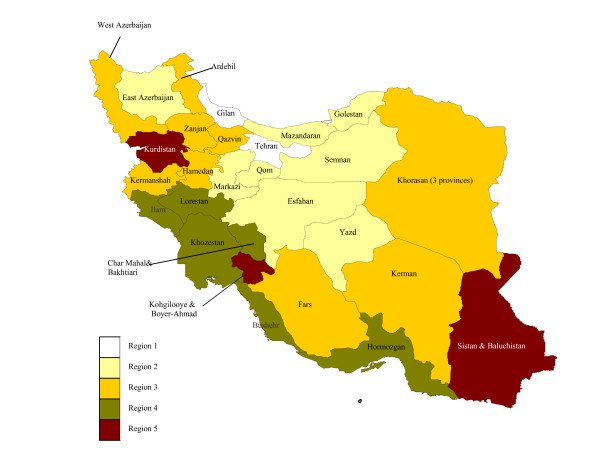
**Classification of Iranian provinces based on levels of mortality and fertility, Statistical Centre of Iran 2000**. Region 1 includes provinces with the lowest mortality and fertility and Region 5 consists of provinces with the highest mortality and fertility levels.

**Figure 12:**
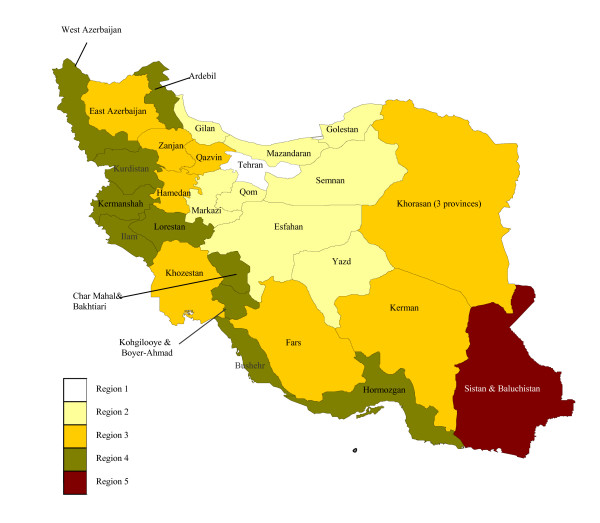
**Modified classification of Iranian provinces based on socioeconomic status (GDP/capita and % literacy)**. Region 1 includes provinces with the lowest levels of socioeconomic indicators (literacy rate and GDP per capita) and Region 5 consists of provinces with the highest levels of socioeconomic indicators.

The Statistical Centre of Iran has grouped provinces into five regions based on their mortality (particularly child mortality) and fertility rates [26]. Figure 11 shows the map of Iran based on this classification. The first region includes provinces with the lowest levels of mortality and fertility, and the fifth region consists of provinces with the highest mortality and fertility levels. It has been claimed that these five regions correspond closely to a classification of the level of development from highest to lowest. However, this classification is based only on these two demographic indicators, and does not adequately reflect the social and economic development of provinces. We have prepared an alternative classification of the socioeconomic development of provinces based on literacy and GDP per capita (see Figure 12).

Our study suggests that there are important variations in child mortality (_5_q_0_) and adult mortality (_45_q_15_) across the country. We also found a close correlation between child mortality and socioeconomic status as measured by GDP per capita (except for the oil producing provinces) and literacy. However, for adult mortality this association is less obvious.

Sistan and Baluchistan province, which is located near to the Afghanistan-Iran-Pakistan borders (AIP region), has the highest level of mortality. The health status of the population living in this area, particularly children and women, is known to be relatively poor [41]. Provinces with a high literacy rate and GDP per capita (e.g. Tehran and Esfahan) tend to have low levels of mortality, confirming similar findings elsewhere.

The three oil producing provinces, Kohgilooye and Boyer-Ahmad, Khuzestan and Bushehr rank first to third among provinces according to GDP per capita. However, Kohgilooye and Boyer-Ahmad province is classified as a "low development" province [42]. About 55% of the population of the province live in rural areas and also this province has a comparatively high nomadic population (86,677 in 2001) [43]. While the Iranian government is working to improve the situation of the population in rural areas, there is still a gap in mortality levels between the rural and urban areas. Part of this difference might be due to the fact that the revenues of the Iranian government from exporting oil products are spent disproportionately on development projects in urban areas than in rural areas [44].

It is important to remember that for the larger provinces in particular, socioeconomic status, and hence mortality patterns, can vary substantially. This is likely to be the case for the more urbanized provinces such as Khorasan, East Azerbaijan, Fars and Kerman. The extent to which the nomadic population form part of the provincial total will also affect the distribution of socioeconomic status. These variations need to be borne in mind when interpreting the strength of the relationship with mortality at the provincial level.

Similar to results from previous studies that have demonstrated a high correlation between child mortality (_5_q_0_) and socioeconomic status (e.g. literacy), our findings also revealed a strong relationship between the level of child mortality and literacy as a key socioeconomic indicator [45]. However, for adult mortality this association is more complex. Non-communicable diseases, such as cardiovascular diseases, or injury are the most important causes of adult deaths and are related to multiple risk factors. As a result, the association between the risk of adult death and socioeconomic factors is not straightforward. This is not the case with infectious diseases which have a more direct association with socioeconomic status.

This study was based on official data from the death registration system operated by the Iranian MOH&ME (Deputy of Health). The coverage of this death registration system has increased in several provinces of Iran over the past five years. Nevertheless, the completeness of data on death registration varies among provinces. Detailed population data by age and sex from a recent census were not available for several large cities, and hence population estimates for urban areas are uncertain. To estimate child mortality (_5_q_0_), we used data on _1_q_0 _since there is no information on _5_q_0 _by province. Since the Iranian MOH&ME's death registration system (Deputy of Health) was not operational in Tehran province (12 million population), we have had to use data on death registration from the Iranian MOH&ME (Deputy of Research and Technology) in 2001 for this province, with known limitations.

In this study, to evaluate the completeness of data on death registration among adults, the Brass Growth Balance Method has been used. The method has several limitations. First, it might be that the assumption of a linear relationship between the partial birth rate and the partial death rate is violated. This could be due to several reasons such as misreporting of age, particularly at advanced ages [34]. When these points are excluded, different estimates of completeness will be obtained. A second source of bias for this method is that the completeness of death registration might vary by age which cannot be assessed from this method [30]. Perhaps most importantly, the method is sensitive to violation of the assumption that the population is stable and closed to migration. This is very rarely the case [30]. In addition, the method is highly sensitive to the estimated slope of the regression line of partial birth and death rates, which in turn depends on the statistical rigour of the method used to estimate the regression model.

Results of the Brass Growth Balance method for some provinces were considered implausible. It is highly likely that migration between provinces, which is non-negligible [46], and under-enumeration of the population data for these provinces were the main reasons for this. There is thus uncertainty around our estimates of completeness of death registration based on this method. Where the method yielded estimates of incompleteness that were relatively high (60–80%), we have accepted that as plausible based on other comparative data and information about the socioeconomic development of provinces.

These adjustments and assumptions were necessary in order to estimate differential mortality in Iran. While this information is undoubtedly useful for health development policies in Iran, the limitations noted above suggest caution in interpreting our findings, particularly small differences between provinces. Further research is necessary to confirm our findings once better provincial mortality data become available.

## Conclusion

This study has used routine data on mortality to estimate the level of mortality for provinces of Iran. While we have identified important differences in survival among provinces, the extent of variation is lower than observed in other developing countries where such studies have been carried out. The various patterns of provincial mortality are closely related to the level of socioeconomic development of provinces. In particular, we found that literacy is a good predictor of the level of mortality in those provinces where indirect demographic methods to adjust the data were inapplicable. While the completeness of data on death registration by the Iranian MOH&ME is reasonable (≥ 75%) in the majority of provinces, further efforts are needed to improve the quality of data on mortality and also to extend the death registration system to include Tehran province.

## Competing interests

The author(s) declare that they have no competing interests.

## Authors' contributions

AK: Contribution to conception and design; acquisition, analysis and interpretation of data; drafting of manuscript.

RT: Contribution to conception and design; interpretation of data; critical revision of manuscript.

MN: Contribution to conception and design; acquisition of data.

ADL: Contribution to conception and design; interpretation of data; critical revision of manuscript.

All authors have read and approved the final manuscript.

## Funding

The Iranian government has provided a scholarship to support Mr. Khosravi's doctoral studies in Australia.
